# A comprehensive insight into functional profiles of free-living microbial community responses to a toxic *Akashiwo sanguinea* bloom

**DOI:** 10.1038/srep34645

**Published:** 2016-10-05

**Authors:** Caiyun Yang, Yi Li, Yanyan Zhou, Xueqian Lei, Wei Zheng, Yun Tian, Joy D. Van Nostrand, Zhili He, Liyou Wu, Jizhong Zhou, Tianling Zheng

**Affiliations:** 1 Research Center of Bioenergy and Bioremediation, College of Resources and Environment, Southwest University, Beibei Dist., Chongqing, China; 2 State Key Laboratory for Marine Environmental Science and Key Laboratory of the Ministry of Education for Coastal and Wetland Ecosystems, School of Life Sciences, Xiamen University, Xiamen 361005, China; 3 College of Life Sciences, Henan Normal University, Xinxiang, China; 4 Institute for Environmental Genomics and Department of Microbiology and Plant Biology, University of Oklahoma, Norman, OK 730722, USA; 5 Earth Sciences Division, Lawrence Berkeley National Laboratory, Berkeley, CA 94720, USA; 6 School of Environment, Tsinghua University, Beijing 100084, China

## Abstract

Phytoplankton blooms are a worldwide problem and can greatly affect ecological processes in aquatic systems, but its impacts on the functional potential of microbial communities are limited. In this study, a high-throughput microarray-based technology (GeoChip) was used to profile the functional potential of free-living microbes from the Xiamen Sea Area in response to a 2011 *Akashiwo sanguinea* bloom. The bloom altered the overall community functional structure. Genes that were significantly (*p* < 0.05) increased during the bloom included carbon degradation genes and genes involved in nitrogen (N) and/or phosphorus (P) limitation stress. Such significantly changed genes were well explained by chosen environmental factors (COD, nitrite-N, nitrate-N, dissolved inorganic phosphorus, chlorophyll-a and algal density). Overall results suggested that this bloom might enhance the microbial converting of nitrate to N_2_ and ammonia nitrogen, decrease P removal from seawater, activate the glyoxylate cycle, and reduce infection activity of bacteriophage. This study presents new information on the relationship of algae to other microbes in aquatic systems, and provides new insights into our understanding of ecological impacts of phytoplankton blooms.

Phytoplankton are the most important primary producers in marine systems and can accumulate to bloom proportions from eutrophication, especially in coastal waters. Blooms dramatically change seawater systems through their efficient autotrophic processes and the exudation of dissolved organic matter^1^, followed by changes in the bacterial^2^,^3^,^4^,^5^, archaeal^6^, viral^7^,^8^ and other microbial communities.

In the phytoplankton bloom area, bacterial abundance increases greatly^4^,^9^, especially species that consume organic matter released by the algae. Virioplankton also increase following increases in bacterial abundance, particularly during peaks in bloom activity. Through these changes to the microbial communities, blooms would eventually affect biogeochemical cycling of elements such as C, N, P and Fe in the marine system^10^,^11^,^12^.

Studies of the influence of phytoplankton blooms on marine ecosystems have primarily focused on the changes of other microbial communities as revealed by 16S rDNA clone libraries^13^, polymerase chain reaction-denaturing gradient gel electrophoresis^14^,^15^, flow cytometry^16^, terminal restriction fragment length polymorphism^13^, fluorescence *in situ* hybridization^17^,^18^, metatranscriptomics^19^, and high-throughput sequencing^20^,^21^. These methods mainly focus on the microbial community instead of their functional potential, except the metatranscriptomics which could be used to investigate the real time function fluctuation based on RNA. These studies confirmed increased bacterial abundance during blooms and subsequent increases in bacteriophage abundance. And there are some interesting findings based on metatranscriptomic analysis. Viral transcription was much higher in the post-bloom environment than during bloom, and bacterioplankton within the bloom had greater upregulation of genes for organic acid utilization and increasing cell surface adhesiveness^19^. Compared to when in monocultures, the dominant dinoflagellate expresses toxin biosynthesis related genes at a higher level in the presence of competitors, predators and prey^22^. It seems that viruses might have less activity and bacteria have enhanced substrate utilization levels during bloom.

The influence of phytoplankton bloom on the biochemical process in the water system is limited, except the sulfur cycle. Phytoplankton in ocean plays an important role on global sulfur cycle by producing massive quantities of dimethylsulfoniopropionate (DMSP)^23^ and promoting specific bacteria species for DMSP turnover, e.g. Ro*seobacter*^24^. What’s more, our former study^25^ (based on Illumina sequencing of 16S rDNA) showed the bacterial diversity decreased, species evenness increased, the bacterial community structure significantly changed, and many bacterial species were stimulated in this bloom, e.g. SAR86 and SAR116 clades and the AEGEAN-169 marine group. Despite these findings, there is still a lot to understand in regards to the functional changes in the microbial community during a bloom.

To obtain a more comprehensive understanding of changes in community function, functional gene arrays (FGAs), microarrays that contain functional gene probes, can be used to simultaneously detect key genes involved in multiple microbial functional processes. The most comprehensive FGAs are the GeoChip arrays. GeoChip 4.2 contains 107,950 probes for genes involved in cycling of C, N, S, P and metals, virulence and antibiotic resistance, the biodegradation of environmental contaminants, stress responses^26^, fungi, pathogens and soil beneficial genes. GeoChips have been successfully used to analyze microbial communities in several marine environments including marine sediments^27^, marine basalt^28^, deep sea hydrothermal vents^29^, the Gulf of Mexico during the 2010 oil spill^30^, mangroves^31^, and coral mucous^32^.

In this study, we used the GeoChip to functionally profile free-living microbial communities in response to an *A. sanguinea* bloom. Specifically, we aimed to address the following questions: (i) does the bloom significantly change the microbial community functional potential? (ii) what functional processes are influenced by the bloom? and (iii) are there specific correlations between the *A. sanguine* population and the functional potential of the associated microbial communities? Our results showed that this bloom significantly altered overall community functional structure and promoted organic carbon degradation, ammonium accumulation and decreased P removal; there were statistical correlations between *A. sanguine* abundance and the functional potential of the microbial community. This study provides new insight into our understanding of the ecological impacts of phytoplankton blooms on microbial communities.

## Experimental Procedures

### Study sites and sample collection

**Figure 1:**
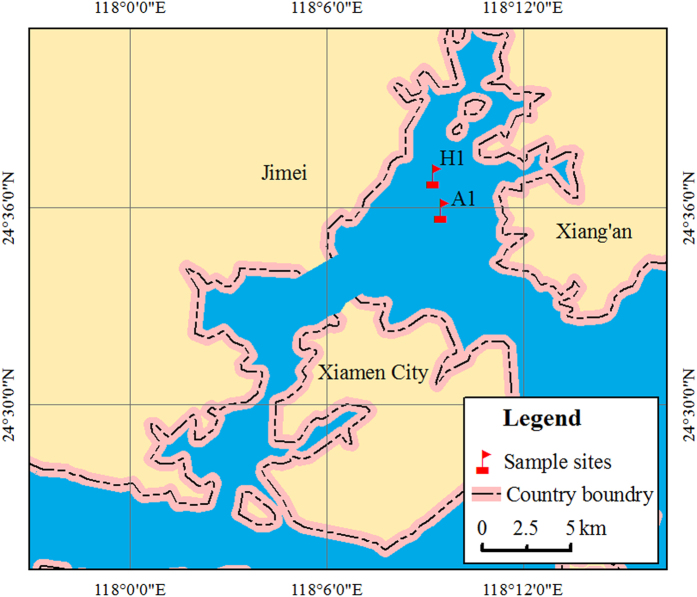
Location of sampling sites. This map was created based on ArcGIS (Version 10, http://www.esri.com/software/arcgis/arcgis-for-desktop) by CYY.

Samples were collected along the Xiamen coast at sites within (A1; N 24°35′53.40′′, E 118°9′29.67′′) or near (H1; N 24°36′56.31′′, E 118°9′15.92′′) the *A. sanguinea* bloom (Fig. 1), during the summer of 2011. Samples were collected each day from 31 July to 5 August as described previously^25^. Briefly, three replicate water samples (20 l) were collected from each site and filtered through 5-μm diameter pore-size filters (Millipore, US) to remove *A. sanguinea* and other large algae. The total free-living bacteria in the filtrate were then collected with 0.22-μm filters (Millipore, US) and stored at −70 °C until analysis.

### DNA extraction, amplification and labeling

For RNA and proteins, sampling procedure (e.g., filtration) could easily induce their expression change^33^. Experiments based on DNA are more stable than that of RNA and proteins, so DNA analysis was focused on in this study. High molecular weight DNA was extracted from filters using a previously described phenol–chloroform–isoamyl alcohol (25:24:1) extraction method^4^ and resuspended in sterile water. DNA quality and quantity was assessed using a spectrophotometer (ND-1000, Nanodrop Inc., DE, U.S.) and a PicoGreen assay^34^, respectively. An aliquot of DNA (25 ng) from each sample was amplified using a TempliPhi kit (GE Healthcare, Piscataway, NJ, U.S.) with a modified buffer^35^, and the amplified DNA (~1 μg) from each sample was labeled and then purified for hybridization^36^,^37^. Control samples from 1 August did not contain enough DNA to perform hybridization, so those samples were removed.

### GeoChip hybridization and data processing

GeoChip 4.2 was used in this study and sample preparation and hybridization were performed as previously described^31^. After hybridization, the arrays were scanned using an MS200 Microarray Scanner (NimbleGen) and images were processed as previously described^31^,^38^,^39^. Spots were scored as positive if the signal-to-noise ratio (SNR) was ≥2.0 and the CV of the background was <0.8. The probes that appeared in only one of the three replicates were removed as noise.

Hybridization data is available at the Institute for Environmental Genomics, University of Oklahoma (http://ieg2.ou.edu/NimbleGen/batch_upload.cgi) and the data was processed as previously described^7^. The relative abundance of each probe signal was then multiplied by the mean of the sums of the original signal intensity for all probes in each sample. All statistical analyses were performed in R^40^. Dissimilarity tests between the bloom and control areas were based on the Bray-Curtis dissimilarity index using analysis of similarities, and the Bray-Curtis dissimilarities among samples using Adonis, ANOSIM and MRPP^36^, and multi-response permutation procedures^27^. Monte Carlo permutation was used to test statistical significance and a significance level of *p* < 0.05 was adopted for all comparisons. Detrended correspondence analysis (DCA) was used to compare the different functional gene communities, and canonical correlation analysis (CCA) was used to link microbial communities to environmental variables. Variation partitioning analysis (VPA) was conducted to examine the contribution of environmental factors in influencing functional gene in the CCA analysis. R statistics were performed running the Vegan (v. 1.15-1)^41^ and agricolae package. Hierarchical clustering was performed with CLUSTER 3.0 with uncentered correlations and the complete linkage for both genes and samples; trees were visualized with Java Treeview.

## Results

The A1 and H1 were bloom and control site, respectively (Fig. 1). The water was distinctly dark in A1 because of the high density of *A. sanguinea*, especially on sampling day 2 and 5. H1 was beside A1 and there was no bloom phenomenon during sampling in this site. The measurement of water parameters (e.g. phytoplankton species identification, cell counts, concentration of chlorophyll *a*, dissolved inorganic phosphorus, nitrate, and so on) were determined as previously described^25^. Based on the algal density dynamics (Table S1), 31 July through 4 August were during the bloom (B); peak algal densities occurred on 1 and 4 August (bloom peaks; BP1 and BP2); and 5 August was after the bloom (AB).

A total of 14171 probes for 575 genes were detected across all samples, including: 1470 probes (39 genes) for C cycling, 1108 probes (17 genes) for N cycling, 196 probes (3 genes) for P cycling, 492 probes (11 genes) for S cycling, 2578 probes (45 genes) for stress response, 85 probes (32 genes) for bacteria phage and 594 probes (13 genes) for virulence (585, 6 and 3 probes from bacteria, fungi and archaea, respectively).

### Total overall functional diversity and structure of free-living microbial communities

**Figure 2:**
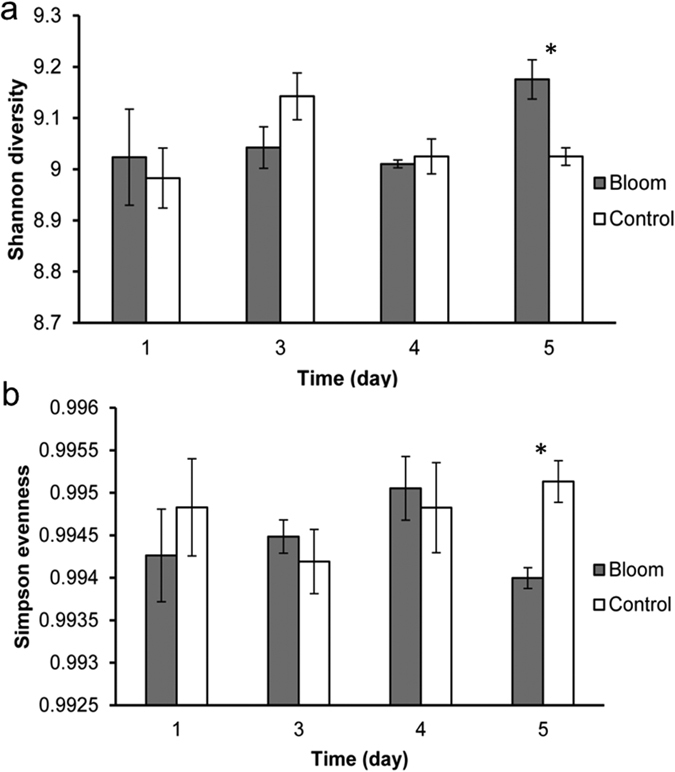
(**a)** Shannon diversity and (**b**) Simpson evenness of total functional genes for bloom and control samples on day 1, 3, 4 and 5. Day 5 was bloom peak 2 (BP2). *p < 0.05; the error bars represent standard errors.

The number and relative abundance of functional genes detected were used to calculate the Shannon–Weiner index (H’) and the Simpson Evenness index (E). For most days, neither the difference in the overall microbial functional diversity (Fig. 2a) nor the evenness (Fig. 2b) between the bloom and control areas was significant (*p* > 0.05). However, on BP2, the functional gene diversity in the bloom area was significantly (*p* < 0.05) higher than that in the control area, while evenness (Fig. 2) was significantly (*p* < 0.05) lower than in the control area. The greatest percentage of unique genes (18.1%) was found in the day 1 bloom samples. Days 3, 4 and 5 had 10.2, 14.8 and 16.5% unique genes (data not shown).

**Figure 3:**
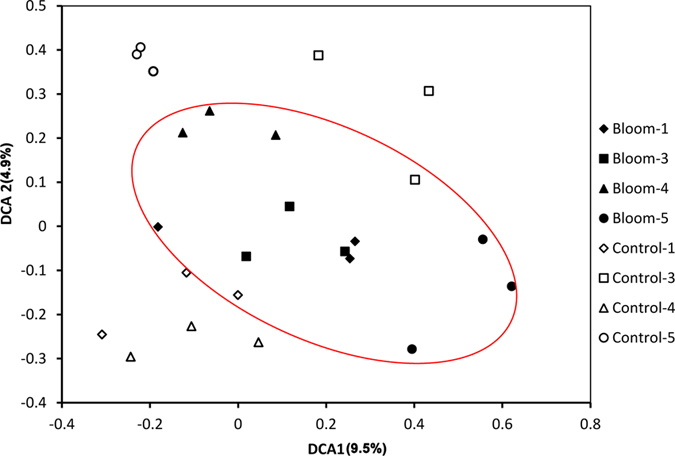
Detrended correspondence analysis of functional genes.

Dissimilarity tests (Table 1) showed there were significant (*p* < 0.01) differences in the functional gene structures of the bloom and control areas based on MRPP analysis on days 1, 3, 4, and 5. And the difference for the total was greater since the p values were all less than 0.05 based on Adonis, Anosim and MRPP analyses. So, the differences were more pronounced between bloom and control areas than over time. The DCA ordination plot (Fig. 3) shows bloom samples grouped in the middle, while the control samples were scattered around the bloom samples, suggesting that the functional gene composition was more similar or stable within the bloom.

### Significantly changed functional genes in response to bloom

**Figure 4:**
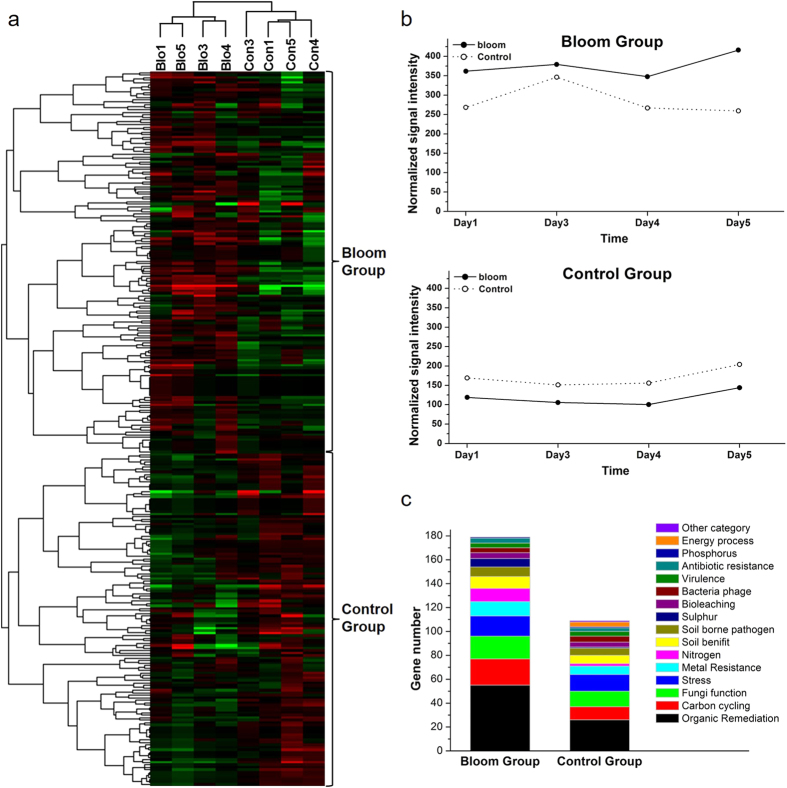
Hierarchical cluster analysis of functional genes from probes with significantly different abundance between bloom and control samples (**a**). Red indicates signal intensities above background (black), while green indicates signal intensities below background. Brighter red or green coloring indicates higher or lower signal intensities. Two major groups were observed. (**b**) The sum of signal intensities for genes from bloom and control sites in groups 1 and 2, (**c**) and the number of genes detected from each gene category in groups 1 and 2.

Based on the functional genes detected, functional potential of microbial communities within bloom samples were significantly (*p* < 0.05) different from control samples (Table 1), comparison was then calculated by T-test among all samples (data not shown). The abundance of 288 genes (1260 probes) were significantly (*p* < 0.05) changed during the bloom. Hierarchical cluster analysis of these 288 genes indicated that samples were well separated by site condition (Fig. 4a).

Based on the functional gene cluster results, two large groups were observed (Fig. 4a). The two groups represented genes with higher abundance either within the bloom (referred to as the Bloom Group, 179 genes) or the control site (referred to as the Control Group, 109 genes) (Fig. 4a,b). There were more functional genes with high abundance in bloom samples. The groups mainly contained organic remediation, carbon cycling, fungi function and stress related genes (Fig. 4c), and the gene numbers of these categories were much higher in the Bloom Group than in Control Group. The most abundant genes were mainly involved in organic carbon degradation: 17 (bloom) and 9 (control) from carbon degradation by bacteria, 13 (bloom) and 7 (control) genes from carbon degradation of fungi, 38 (bloom) and 16 (control) genes from aromatic carbon degradation. Gene abundance changes followed similar patterns for both bloom and control samples in Control Group, while these changes varied for bloom and control samples in Bloom Group (Fig. 4b).

There were more N cycling related genes in Bloom Group (11 genes) than Control Group (2 genes), and the 11 genes were involved in denitrification (*nosZ*, *nirS*, *nirK* and *narG*), assimilatory N reduction (*nasA*, *nir* and *nirA*), dissimilatory N reduction (*napA* and *nrfA*), nitrification (*amoA*) and nitrogen fixation (*nifH*); the 2 genes were involved in ammonification (*gdh* and *ureC*). The signal intensity changing pattern of those genes was shown in the simplified N cycling diagram (Fig. S1). This suggests the bloom community may have an accelerated N cycle stimulating the utilization of nitrate and nitrite, e.g., ammonification, N fixation and denitrification processes. Bloom Group also had more S cycling related genes (7) than Control Group (1), including genes for adenylylsulfate reductase, sulfide oxidation, sulfite reductase and sulphur oxidation. Control Group had increased *dsrB* for sulfite reductase.

Bloom Group had more stress genes related to N limitation (1 to 0) while Control Group had more related to P limitation (1 to 5). Bacteriophage genes involved in lysis and replication were high in Control Group (5 genes) than Bloom Group (2 genes), indicating the bloom might decrease viral infection of bacteria.

### Relationship between significantly changed functional genes and environmental parameters

**Figure 5:**
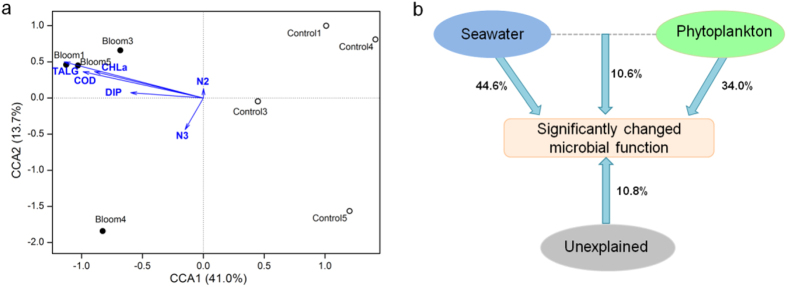
CCA of significantly changed gene data with selected environmental variables (**a**) and partial CCA-based variation partitioning analysis (VPA) (**b**). The selected environmental variables include chemical oxygen demand (COD), nitrite nitrogen (N2), nitrate nitrogen (N3), dissolved inorganic phosphorus (DIP), chlorophyll *a* (CHLa) and total algal density (TALG).

The 288 significantly changed genes represented the main microbial functional responses to this bloom. To test how environmental variables contributed to the functional gene differences, CCA was performed using all 288 genes and measured seawater and phytoplankton properties (Fig. 5a), including chemical oxygen demand (COD), nitrite-nitrogen (N2), nitrate-nitrogen (N3), dissolved inorganic phosphorus (DIP), chlorophyll *a* (CHLa) and total algal density (TALG).

In the CCA ordination plot (Fig. 5a), the first two canonical axes explained 41.0% and 13.7% of the constrained variation, respectively, in the significantly changed functional genes. The bloom and control samples were well separated along the first canonical axis (CCA1). The projection of environmental variables indicated that bloom samples (especially for the day1, 3, and 5 when the algal density was higher than day 4) showed a positive correlation with DIP, N3, TALG, CHLa and COD, while control samples were negatively correlated with these variables. The length of the arrows indicated that the impacts of TALG, CHLa and COD were greater than those of other variables.

Variation partitioning analysis (VPA) was conducted to test the seawater properties (COD, N2, N3 and DIP) and phytoplankton (CHLa and TALG) in influencing the significantly changed genes in the CCA analysis (Fig. 5b). The seawater and phytoplankton variables explained 44.6% and 34.0% of the variation in the genes, respectively, and their interaction explained 10.6%, leaving 10.8% unexplained.

### Genes linked to the *Akashiwo sanguinea* community

**Figure 6:**
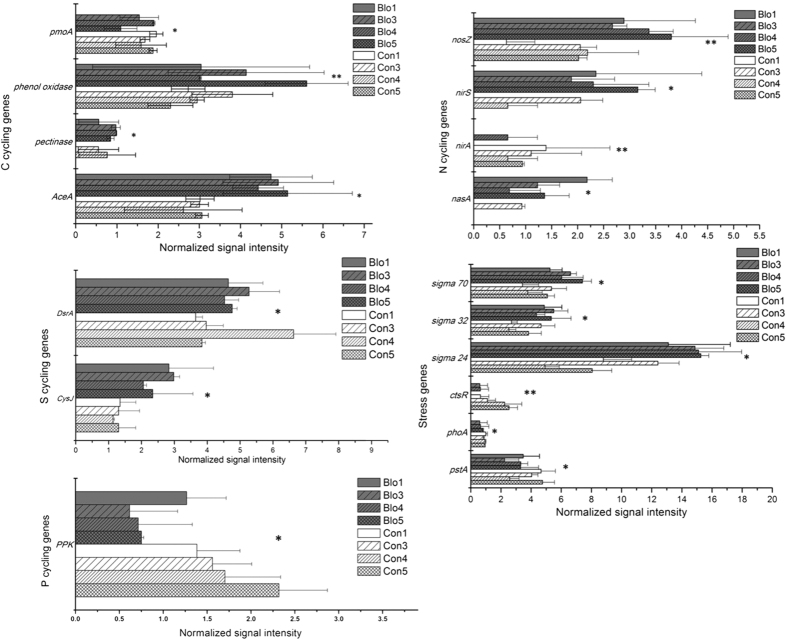
A subset of significantly changed genes in the bloom compared to the control site. *0.01 ≤ *P* < 0.05; **p < 0.01.

Among the significantly changed genes, 67 showed significant (*p* < 0.05) correlations with *A. sanguinea* density; genes within C, N, S, P cycling and stress categories are shown in Fig. 6. The bacterial C degradation genes (*Ace*A-isocitrate lyase, pectinase and phenol oxidase) showed significantly positive correlations with *A. sanguinea* while a gene for methane monooxygenase showed an inverse correlation. Four genes (*nasA*, *nirA*, *nirS* and *nosZ*) for assimilatory N reduction and denitrification were significantly correlated with *A. sanguinea*. The positive correlation with *nirS* and *nosZ* suggest that this bloom enhanced the denitrification potential of the free-living microbes. *CysJ* and *dsrA* for sulfite reductase showed higher relative abundances in bloom samples and had significantly positive correlations with *A. sanguinea*. This suggests that the sulfite reduction potential was enhanced during the bloom. The P cycling gene *ppk* had a lower relative abundance in the bloom and showed an inverse correlation with *A. sanguinea*.

The stress response genes, *pstA* and *phoA*, for phosphate limitation, showed significantly negative correlations with *A. sanguinea* abundance. The heat shock genes-sigma 24, 32 and 70 showed significantly positive correlations with *A. sanguinea* while *ctsR* showed an inverse correlation, and the marine ecological function of these genes are still unclear. Bacteriophage related genes for replication and lysis showed a significantly inverse correlation with *A. sanguinea*, indicating the bloom might weaken potential of viruses to infect bacteria.

## Discussion

Previously, we revealed how the free-living bacterial community responded to an *Akashiwo sanguine* bloom based on 16S rDNA sequencing^25^. This is a parallel study, examining functional gene changes in the communities examined in the earlier paper.

In this study, the ~10,000 detected probes covered a wide range of functional gene categories, indicating the community contained a high degree of microbial functional potential. This laid the foundation for the community’s resilience to natural disturbances.

### Bloom doesn’t impact the functional potential of free-living microbial communities as greatly as expected

*A. sanguinea* blooms in the Xiamen Sea Area appeared for the first time in 2008^4^ and have subsequently frequently occurred. Marine bacterial communities have been reported to be greatly influenced by *A. sanguinea* blooms^4^,^25^, including the bacterial abundance and community structure. But these studies were limited in their ability to reveal the influence of the blooms on bacterial and any other microbial functions. We hypothesized that the bloom would have a significant impact on free-living microbial function as compared to those in the control site. The overall community structures showed significant differences (Table 1) which were more pronounced between bloom and control areas than over time. But few individual genes/gene groups showed significant differences. Less than 10% of the detected probes were significantly different between the two sites. Most of the bloom-enriched genes were involved in organic matter degradation, which is unsurprising since high concentrations of phytoplankton can produce abundant organic matter from sunlight and inorganic matter^42^.

The microbial community functional structure appeared to be more stable within the bloom compared to the control, consistent with our previous results which based on bacterial community^25^. This could result from both environmental conditions and the bloom itself^43^, providing relatively stable environmental conditions necessary for bloom formation and sustainability^44^,^45^,^46^. In addition, the abundant algae within a bloom release organic matter^42^,^47^, which serves as a C source for the microbes within the bloom and influences environmental conditions. COD, N2, N3, DIP and TALG appeared to be strong drivers in selecting the microbial communities.

### N and P important for bloom communities

Microbial communities within the bloom were enriched in N and P cycling genes, suggesting these elements are particularly important in this environment. Changes in many genes suggested the N cycling potential was accelerated in the bloom, especially for utilization of NO_2_^−^ (denitrification) and NO_3_^−^ (dissimilatory reduction of nitrate to ammonium), nitrogen fixation, and ammonium production. N limitation stress genes were among the bloom enriched genes, suggesting that N consumption in this field was quite large and that extraneous N input and biological N fixation did not meet demand. N limitation could be one reason for the subsequent bloom termination. In addition, the bloom may have increased the transfer potential of nitrate to N_2_ by enhancing the intensity of denitrification related genes (*nosZ*, *nirS*, *nirK narG*). The total nitrogen dynamic in mixed cultures of algae and other microorganisms hasn’t been reported. One report indicated that denitrification increased and total nitrogen concentration decreased 25.97–28.16% in *Microcystis aeruginosa* cultures^48^. Additional research into the influence of blooms on N cycling is needed.

Little is known about nutrient utilization by *A. sanguinea*. Raphael *et al.*^49^ reported that the uptake rates for ammonium are much higher than for NO_3_^−^ in single substrate experiments. Similar results were obtained for N-specific uptake rates of natural assemblages dominated by *A. sanguinea* in California^49^, suggesting ammonium is a preferred N source of *A. sanguinea.* The production potential of ammonium from N_2_ and nitrate might be enhanced in the free-living microbial community by enhancing the intensity of *nifH*, *nasA*, *nir*, *nirA, napA* and *nrfA* genes, which might have contributed to the duration of this bloom. This potential beneficial effect for the algae from the microbial community is in contrast to other studies that have observed an inhibition or lethality of bacteria and phage towards algae during blooms^42^,^50^,^51^,^52^,^53^,^54^.

The control site contained more P limitation stress genes, suggesting P limitation may have prevented bloom formation in the control site. In addition, there was more DIP in the bloom^40^. P is required for the fast growth and bloom formation of *A. sanguinea*^25^. Polyphosphate kinase *(ppk)* catalyzes the formation of polyphosphate (polyP), a process by which microbes could remove P from water^55^. The decrease in *ppk* as *A. sanguinea* increased may be conducive to the bloom duration.

### Novel impacts associated with the bloom

Changes in some of the functional genes detected in this study suggest that the bloom had surprising influences on the microbial communities.

#### Bacteria might be protected from phage

There were fewer lysis and replication related genes of bacterial phage in the bloom suggesting that phytoplankton blooms might reduce viral infections in bacteria. Studies on the infection dynamics of bacteriophage influenced by algal blooms are limited, but Maranger *et al.*^56^ found a disequilibrium between phage and bacterial growth and abundance during a spring ice algal bloom. During the bloom, the virus-to-bacteria ratios decreased although virus abundance increased. The authors speculated that proliferation of phage-resistant bacteria was one reason. Another study of a mesotrophic lake in Germany^57^ found that phage-induced mortality was lower during phytoplankton blooms (<11%) than after a bloom (18–21%). These results suggest that the bloom environment protects bacteria from bacterial phage. Maintaining a high abundance of bacteria may help to avoid accumulation of the massive amounts of organic matter produced by blooms, and may be an ecological strategy to maintain balance.

#### Bacteria activated glyoxylate cycle for isocitrate metabolism.

The *AceA* gene which encodes isocitrate lyase showed a significant positive correlation with *A. sanguinea*, indicating the glyoxylate cycle was more active in bloom-bacteria than in non-bloom bacteria. Rinta-Kanto *et al.* found bloom bacterioplankton transcribed more copies of genes for organic acid utilization, but transcripts for isocitrate dehydrogenase were less abundant in the bloom transcriptome^19^. It’s possible that the energy storage for bacteria within the bloom was enough, so more-than-usual isocitric acid entered into glyoxylate cycle by isocitrate lyase catalyzing, instead of entering tricarboxylic acid cycle by isocitrate dehydrogenase catalyzing (the main energy production process). And this resulted in higher net carbon assimilation.

## Conclusion

We applied the GeoChip to the study of a phytoplankton bloom and found that the *A. sanguinea* bloom did not change the free-living microbial function potential as greatly as expected. Community differences were more pronounced between bloom and control areas than over time. Most significantly changed genes showed an increase in intensity within the bloom, especially for the genes related to carbon degradation. N or P availability appeared to be important for both start and termination of the bloom. The microbes in the bloom appeared to contribute to bloom duration by promoting ammonium accumulation and decreasing the competitive adsorption of biological Pi removal from water. In addition, the bloom may reduce infection activity of bacteriophage by decreasing the intensity of replication and lysis related genes.

Although this study was based on DNA, which provides indirect ecological evidence of functional potential, results were consistent with transcription studies in this field. Changes in gene abundance suggest that the bloom enhanced microbial ammonia accumulation and the transfer of nitrate to N_2_, reduced infection activity of bacteriophage, and that isocitrate was actively shuttled through the glyoxylate cycle in bacteria. These findings provide new research directions for the study of bloom dynamics and provides a deeper insight into the relationship between bloom algae and other microbes in marine systems. Continuing research is focused on RNA studies.

## Additional Information

**How to cite this article**: Yang, C. *et al.* A comprehensive insights into functional profiles of free-living microbial community responses to a toxic *Akashiwo sanguinea* bloom. *Sci. Rep.* **6**, 34645; doi: 10.1038/srep34645 (2016).

## Supplementary Material

Supplementary Information

## Figures and Tables

**Table 1:** Dissimilarity tests of functional gene structure between bloom and control areas.

Bloom vs. Control	Adonis		Anosim		MRPP	
	F	*p*	R	*p*	δ	*p*
Day1	47.11	0.1	0.407	0.12	0.352	<0.01
Day3	42.77	0.11	0.815	0.1	0.389	<0.01
Day4	39.407	0.08	0.107	1	0.464	<0.01
Day5	37.651	0.1	0.086	1	0.548	<0.01
Total	52.263	<0.01	0.123	0.02	0.08	<0.01
