# Supplementary material for: A comprehensive insight into functional profiles of free-living microbial community responses to a toxic Akashiwo sanguinea bloom
Source: Sci Rep. 2016 Oct 5;6:34645. doi: 10.1038/srep34645 (PMC5050414; doi:10.1038/srep34645)
Supplement: Supplementary Information [file srep34645-s1.pdf]

A comprehensive insights into Functional profiles of free-living microbial community responses to a toxic *Akashiwo sanguinea* bloom

Caiyun Yang[1,2†], Yi Li[2,3†], Yanyan Zhou[2], Xueqian Lei[2], Wei Zheng[2], Yun Tian[2], Joy D. Van Nostrand[4], Liyou Wu[4], Zhili He[4], Jizhong Zhou[4,5,6*], Tianling Zheng[2*]

Table S1 (Yang *et al.*, 2015) Properties of samples during the *Akashiwo sanguinea* bloom in 2011 (Only the samples for comparison (bloom vs. control) are ahown)

Samples	T	Sal	pH	SP	DO	NO_2^-	NO_3^-	NH_4^+	DIN	DIP	N/P	SiO_4^-	COD	CHLa	EI	BACT	*A. sa*	TALG
A1-1	31	24	7.99	2.8	5.25	0.032	0.186	0.009	0.227	0.021	11	0.87	4.66	67.91	4.84	4.60×10^{11}	1.08×10^{5}	1.32×10^{5}
A1-2	31	26	7.97	19.0	4.66	0.029	0.194	0.009	0.232	0.032	7.33	0.84	2.04	51.9	3.32	4.11×10^{11}	2.05×10^{5}	2.36×10^{5}
A1-3	30.8	28	7.89	15.5	4.57	0.030	0.355	--	--	0.026	--	0.95	2.95	23.32	--	9.96×10^{11}	1.55×10^{5}	1.84×10^{5}
A1-4	30	29	7.87	14.2	4.46	0.030	0.296	0.028	0.354	0.020	18.03	0.90	2.01	33.09	3.09	3.11×10^{11}	0.81×10^{5}	0.99×10^{5}
A1-5	30.5	25	7.84	13.8	3.9	0.013	0.117	0.018	0.147	0.010	15.38	0.57	1.89	52.97	0.59	2.86×10^{11}	1.70×10^{5}	1.89×10^{5}
A1-8	31	30	7.82	10.1	3.51	0.026	0.255	0.078	0.359	0.027	13.20	1.02	1.38	21.3	3.00	--	0.10×10^{5}	0.37×10^{5}
H1-1	31	24	7.89	13.6	6.17	0.043	0.183	0.002	0.228	0.012	18.96	0.79	1.45	38.65	0.88	1.77×10^{11}	0.18×10^{5}	0.41×10^{5}
H1-2	31	24	7.87	10.6	--	0.027	0.213	0.026	0.266	0.013	20.87	0.56	0.97	28.13	0.74	1.00×10^{11}	0.08×10^{5}	0.18×10^{5}
H1-3	31	25	7.84	10.4	3.65	0.023	0.186	0.031	0.2393	0.024	10.06	0.68	1.26	31.31	1.59	0.75×10^{11}	0.22×10^{5}	0.35×10^{5}
H1-4	30.5	29	7.78	14.6	3.76	0.015	0.194	0.030	0.2385	0.012	20.24	0.79	1.36	21.58	0.85	1.23×10^{11}	0.63×10^{5}	0.76×10^{5}
H1-5	30.5	29	7.75	15.0	3.73	0.023	0.230	0.040	0.2927	0.009	34.02	0.94	1.04	23.14	0.58	2.38×10^{11}	0.08×10^{5}	0.14×10^{5}
H1-8	31.8	26	7.61	13.0	6.13	0.039	0.375	0.122	0.535	0.050	10.61	1.51	1.14	20.81	6.87	--	0.33×10^{5}	0.36×10^{5}

The sample numbers indicate time (days), 1: 31 July; 2-5: 1-4 August; 8: 7 August. T: temperature, ºC; Sal: salinity,%; SP: suspended particles, mg/L; DO: dissolved oxygen, mg/L; NO_2^-: nitrite nitrogen, mg/L; NO_3^-: nitrate nitrogen, mg/L; NH_4^+: ammonia nitrogen, mg/L; DIN: dissolved inorganic nitrogen, mg/L; DIP: dissolved inorganic phosphorus, mg/L; N/P: ratio of dissolved inorganic nitrogen and DIP; SiO_4^-: silicate mg/L; COD: chemical oxygen demand, mg/L; CHLa: chlorophyll *a*, ug/L; EI: Eutrophication index; BACT: bacterial density, cells/mL; *A. sa*: *Akashiwo sanguinea* density; TALG: total algal density;--: not available.

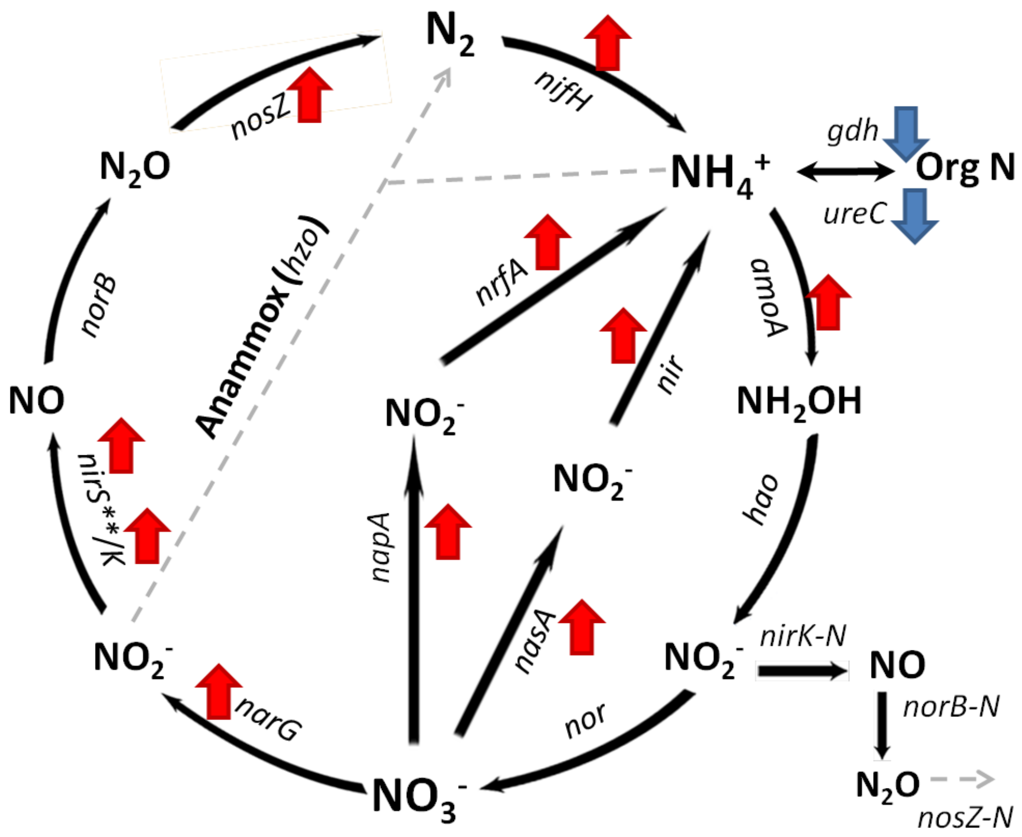

Figure S1 A simplified N cycle diagram with gene signal intensity changes (*P*< 0.05) in the bloom compared to the control site. The red arrows indicates a signal intensity increase while blue arrows indicate a signal intensity decrease.
